# ltrafiltration-based sample preparation and HPLC-UV determination of diclofenac in human plasma samples

**DOI:** 10.55730/1300-0527.3367

**Published:** 2022-01-18

**Authors:** Merve NENNİ, Ayşegül DOĞAN, Mustafa ÇELEBİER, Murat SOYSEVEN, Mustafa Sinan KAYNAK, Hassan Y. ABOUL-ENEIN, Göksel ARLİ

**Affiliations:** 1 Department of Analytical Chemistry, Faculty of Pharmacy, Çukurova University, Adana, Turkey; 2 Department of Analytical Chemistry, Faculty of Pharmacy, Hacettepe University, Ankara, Turkey; 3 Department of Medical Services and Techniques, Yunus Emre Vocational School of Health Services, Eskişehir, Turkey; 4 Department of Pharmaceutical Technology, Faculty of Pharmacy, Anadolu University, Eskişehir, Turkey; 5 Department of Pharmaceutical and Medicinal Chemistry, National Research Center, Cairo, Egypt; 6 Department of Analytical Chemistry, Faculty of Pharmacy, Anadolu University, Eskişehir, Turkey

**Keywords:** Centrifugal filters, diclofenac, high-performance liquid chromatography, HPLC, plasma, ultrafiltration

## Abstract

The sample preparation step is the initial step in pharmaceutical analysis. While ultrafiltration is a well-known technique used in the food and pharmaceutical industries, it has rarely been used to measure the plasma concentration of active pharmaceutical ingredients. This study aimed to analyze diclofenac sodium (DS) in human plasma samples using ultrafiltration-based sample preparation before high-performance liquid chromatography (HPLC) analysis. The advantages and limitations of ultrafiltration-based sample preparation in bioanalysis were evaluated by comparing the results with conventional methods. The precipitating agent was used before ultrafiltration. The analysis was carried on an HPLC-UV system with a C18 column (250 ×4.6 mm, 5 μm) and acetonitrile : phosphate buffer (pH 3.0, 10 mM) (70 : 30 v/v) was used as the mobile phase. The bioanalytical method was validated according to FDA guidelines and applied to spiked samples of DS in commercial human plasma samples. The LOD and LOQ values were 0.006 μg mL^−1^ and 0.020 μg mL^−1^, respectively. The method was linear in the range of 0.025–0.50 μgmL^−1^ with excellent determination coefficients (R^2^ > 0.9991). The findings of this analysis with low LOD and LOQ values and high recovery values with high trueness and precision proved the matrix minimizing the effect of the presented sample preparation technique.

## 1. Introduction

Ultrafiltration is the extraction of macromolecules from micromolecules through membranes. The diameter in the membranes used in ultrafilters can start at 1 nm and go up to 100 nm. Proteins in macromolecule structures can easily be separated selectively by the ultrafiltration method. Many factors affect this method’s capacity, like solute diameter, instrumental configurations, and membrane pore diameter. Membranes are the critical component of ultrafiltration. They consist of organic polymers. Polyamide, polysulfones, and cellulose are often used as polymers. The solution’s passage through the ultrafilter membrane is related to the implemented pressure. The solution’s transfer through the ultrafilter membrane is connected to the implemented pressure. Depending on their size, membranes take part as a selective barrier that controls the transfer of molecules through them. The companies first produced filters with a criterion about molecular size. This criterion is the “molecular weight cutoff.” It explains that molecules of specific sizes are approved to pass through the filters. Afterward, solute-solvent and solute-membrane interactions began to be observed. Membrane modifications have started with the development of extraction technologies. The developments also accomplish the problems of membrane fouling and also increase the separations’ selectivity. Parameters such as pH of a solution, ionic strength, flow rate, and percolation flow, which play a role in transport quality, still need improvement [1]. Suspended solids and solutes with high molecular weight are filtered under pressure while passing through the membrane. In the research and development laboratory, the ultrafiltration method has been applied for years as extraction progress in the purification and concentration of high molecular weight solutions (especially proteins). This unique technique has found itself in production processes, particularly in the food industry and the pharmaceutical industry [2].

High-performance liquid chromatography (HPLC) is still one of the most dominant. When the 2000s were just beginning, a brand new HPLC system with leading technology was designed. It is called ultra-high-performance liquid chromatography (UPLC) [3,4]. As a result of preventing the interference of emerging matrix ingredients and maintaining the durability of the new generation columns for the HPLC, some sample preparation procedures should be applied for human plasma samples before the analysis. The most common sample preparation techniques for bioanalysis are the precipitation of proteins (deprotonation) with some organic solvents [5], liquid-liquid extraction [6], and solid-phase extraction [7]. Ultrafiltration was frequently used in the ‘90s for sample preparation [8, 9]. However, there are few studies using ultrafiltration for this purpose. These studies draw attention to the analysis of free (unbound) API concentrations in plasma [10].

The most commonly prescribed drugs besides antibiotics are NSAIDs. They are used in the case of inflammatory diseases, pain, and fever. DS is a member of NSAIDs and it has antiinflammatory, analgesic, and antipyretic effects. Its availability of it as over the counter in most countries facilitate its accessibility and increases patients’ interest in it. It is frequently preferred for rheumatic cases and joint inflammations after surgical interventions, trauma, and migraine [11]. Similar to other group members, DS binds to plasma proteins at a rate of 99.7% [12]. DS is an active pharmaceutical ingredient having a high bonding capacity to proteins and soluble in water. Thus, the applicability and benefits of an innovative approach using precipitation of proteins combined with ultrafiltration-based sample preparation on bio-pharmaceutical analysis were presented in this study when DS was used as a model molecule. This methodology can be adapted to analyze pharmaceuticals in blood samples regardless of their physical properties.

## 2. Materials and methods

### 2.1. Chemicals

DS and Naproxen sodium (Internal Standard, IS) were supplied from Sigma-Aldrich. Collected via K_2_-EDTA procedure, pooled blank human plasma was bought from EMR chromatography, Turkey. HPLC grade acetonitrile and methanol were supplied from Merck. Furthermore, sodium phosphate dibasic was supplied from Merck. Milli-Q water was supplied from the Barnstead Nanopure™ system of Thermo Scientific.

### 2.2. Apparatus

The LC system (Agilent 1220 Infinity II) is equipped with an Ultraviolet/Visible (UV/Vis) detector, and ChemStation software is used. Hettich UNIVERSAL 320 R centrifuge and Ika Vortex Genius were used during the analysis. Microcon^®^ centrifugal filters (<3 kD) were purchased from EMD Millipore.

### 2.3. Preparing mobile phase buffer of HPLC analysis

1.56 g of sodium dihydrogen phosphate (NaH_2_PO_4_) was dissolved in 1L water to produce 10.0 mM phosphate buffer. The pH of the buffer was maintained to 3.0 using o-phosphoric acid.

### 2.4. Chromatographic conditions

Phenomenex C18 (250 × 4.6 mm, 5 μm) LC Column was utilized for separations. Mobile phase was consist of acetonitrile-phosphate buffer (pH 3.0, 10 mM) (70 : 30 v/v). The flow rate was 1.0 mL min^−1^ in an isocratic elution mode. The injection volume was 20 μL, and the 284 nm wavelength was employed for UV detection.

### 2.5. Ultrafiltration procedure

**Figure 1:**
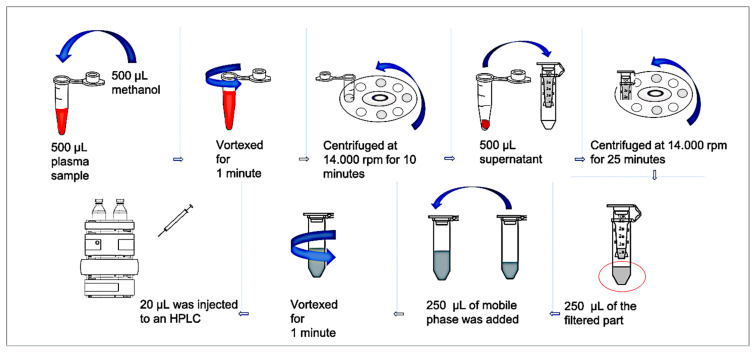
Schematic presentation of the ultrafiltration procedure.

The procedure used in sample preparation: 1) Take 200 μL of plasma samples into an eppendorf tube 2) Add 200 μL of methanol to precipitate the proteins. 3) Vortex for 1 min. 4) Take supernatant into Amicon Ultra-0.5 Centrifugal Filter having 3kDa pores 5) Centrifuge at 14,000 rpm for 10 min. 5) Take 250 μL of the supernatants to an Eppendorf tube. 6) Add 250 μL of mobile phase 7) Vortex for 1 min. 8) Transfer 20 μL of supernatant into HPLC (Figure 1). The final dilution factor was 4x at the end of the ultrafiltration-based sample preparation.

### 2.6. Standard stock solutions

The standard stock solution of DS (1000 μg mL^−1^) and IS (1000 μg mL^−1^) were prepared in methanol. The standard stock solution is stored at 4 °C until the analysis and freshly prepared twice a week. Stock solutions were prepared by diluting the mobile phase to the concentrations to be used, and the ultrafiltration procedure was applied before the HPLC-UV analysis and kept at 4 °C during the analysis and freshly prepared twice a week. Stock solutions were diluted to desired concentrations using the mobile phase, and the ultrafiltration procedure was applied before HPLC-UV analysis.

### 2.7. Preparation of calibration curves

Calibration curves were constructed using the commercially available blank plasma samples. DS and IS stock solutions were spiked in required amounts into the Eppendorf tubes containing filtrated blank plasma samples (250 μL), and the final volume was filled up to 500 μL by adding the mobile phase to obtain final concentrations as follow: 0.025, 0.05, 0.075, 0.113, 0.15, 0.25, 0.50 μg mL^−1^ DS, and 0.15 μg mL^−1^ IS. Peak area ratios of DS/IS were plotted against concentrations of DS.

### 2.8. Analysis of DS spiked plasma samples

The ultrafiltration procedure was applied to the spiked plasma samples containing 0.20, 0.45, and 1.00 μg mL^−1^ DS and 0.60 μg mL^−1^ IS. When the dilution factor (4x) was considered after ultrafiltration-based sample preparation, the final solutions consisting of 0.05, 0.1125, and 0.25 μg mL^−1^ of DS and 0.15 μg mL^−1^ of IS were analyzed by calibration equation, and the results were calculated statistically for six replicates.

### 2.9. Analytical method validation

The developed method has been validated according to FDA guidelines. In addition, the calibration curves, sensitivity, precision, trueness, and selectivity of the method were examined [13].

## 3. Results and discussion

### 3.1. Optimization of the chromatographic conditions

During the optimization process, acetate and phosphate buffers, mobile phases at various ratios, different pH and concentration values of phosphate buffer were tried and the best chromatographic separation with the least retention time in acceptable range was investigated. For this, methanol : water mixture was used firstly, but tailing in the peaks observed. Afterward, acetonitrile : water mixture was used as the mobile phase, and it was observed that the tailing of the peaks decreased and the analysis time was relatively shortened. Then, the analyzes were performed using buffer instead of water in the mobile phase. Thus, sharper peaks were observed using buffer and it was determined that the method was not affected by small pH changes. Finally, DS determination from plasma spiked samples was applied with a C18 (250 × 4.6 mm, 5 μm) column using a mobile phase of acetonitrile: phosphate buffer (pH 3.0, 10 mM) (70 : 30 v/v). The total analysis time was shorter than 7 minutes under the optimum conditions.

### 3.2. System suitability

The system suitability parameters of the developed HPLC method according to FDA bioanalytical method validation were injection precision (<1%), capacity factor (1 < k′ < 10), tailing factor (<1.5), theoretical plate number (>2000). Injection precision relative standard deviation (RSD) value was found as 0.12% in 6 replicate trials. The capacity factor of 6.56, tailing factor of 1.09, and theoretical plate number (Efficiency, N) of 5284 were calculated, indicating that the system suitability of the developed method covers the requirements.

### 3.3. Method validation

#### 3.3.1. Linearity, range, LOQ, and LOD

**Figure 2:**
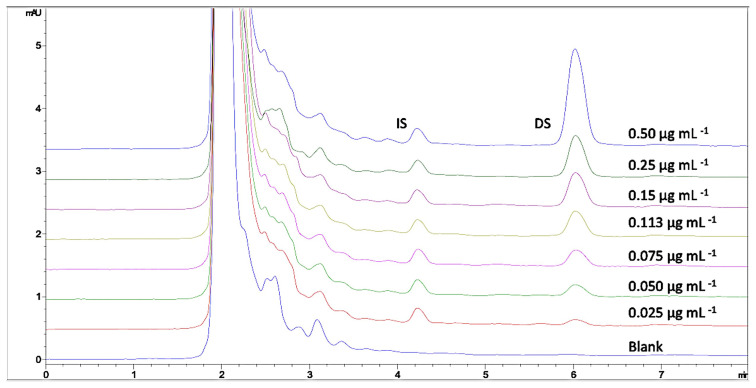
The overlaid chromatograms of blank plasma sample and calibration curve samples (0.025–0.5 μg mL^−1^ of DS and 15 μg mL^−1^ of IS) under experimental conditions: Mobile phase: acetonitrile : phosphate buffer (pH 3.0, 20 mM) (70 : 30 v/v), flow rate: 1 mL min^−1^, injection volume: 20 μL, detection wavelength: 284 nm, IS concentration for calibration curve: 0.15 μg mL^−1^.

The calibration curve was constructed by plotting peak area ratios to the concentration of DS in the range of 0.025–0.50 μg mL^−1^. These concentrations represent 0.1–2.0 μg mL^−1^ of DS for real human plasma samples since the dilution factor was 4x for the ultrafiltration procedure. The regression equation and correlation coefficient calculated were y = 17.1837x + 0.3489 and R^2^ = 0.9991 (y = ax+b; y is the peak area ratio of DS to IS and x is the final concentration of DS after 4x dilution). The chromatograms of blank plasma samples and calibration curves are given in Figure 2. The limit of detection (LOD) and limit of quantification (LOQ) values for DS were calculated according to the signal/noise ratio equal to 3 and 10, respectively. The detected peaks which refer to 0.006 μg mL^−1^ and 0.020 μg mL^−1^ of the final DS concentrations were found as LOD and LOQ, respectively for the proposed methodology (Table 1).

#### 3.3.2. Trueness and precision of the developed method

Three different concentrations of DS (0.0500, 0.1125, and 0.2500 μg mL^−1^) within the linear range as low medium and high concentrations were analyzed in three consecutive days (interday studies) and three times within the same day (intra-day studies). The biases of intra-day and interday studies were between −4.77 and 3.23 which proves the high trueness of the method.

The relative standard deviations of intra-day and interday studies were between 2.86% –5.99% respectively, which were below the FDA’s limits of 15% for bioanalytical analysis. These results indicate that the developed method was accurate and precise. Intermediate precision was also controlled by injection repeatability, and a value of 0.12%, which is below the acceptance criteria of 2%, shows the high precision of the method (Table 2).

#### 3.3.3. Selectivity of the developed method

**Figure 3:**
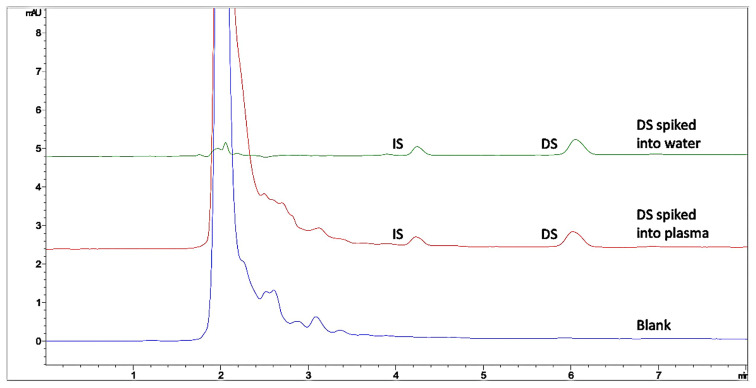
The overlaid chromatograms of blank plasma sample, DS spiked into the water and spiked into plasma samples under experimental conditions. DS concentration is 0.45 μg mL^−1^, and IS concentration is 10 μg mL^−1^ before the extraction procedure.

The selectivity criteria for DS were examined from chromatograms in the presence of plasma. Therefore, chromatograms of blank plasma, DS, and IS spiked into plasma and water were compared (Figure 3). The interference peaks coming from matrix components were tracked to show if they interfere with the peaks of DS and IS. As was expected, the water-soluble matrix components eluted with the dead volume where DS and IS were eluted at 3.20 and 2.21 min.

### 3.4. Analysis of spiked plasma samples

DS spiked plasma samples (0.20, 0.45, and 1.00 μg mL^−1^ DS and 0.60 μg mL^−1^ IS) were prepared as defined in the experimental part. The dilution factor after ultrafiltration-based extraction was 4x, which means the final solutions of the spiked samples contain 0.05, 0.1125, and 0.25 μg mL^−1^ of DS, and 0.15 μg mL^−1^ of IS. These solutions were analyzed using the developed HPLC method, and the results were calculated using the regression equation (Table 1) get from calibration standards. The analysis results showed that the recovery for DS spiked into plasma samples was between 93.11% and 104.92% for three different concentration range.

As seen from the results, the proposed ultrafiltration-based extraction coupled with the developed RP-HPLC method could be used successfully to analyze of DS in human plasma samples.

### 3.5. An overview of the discussion

The ultrafiltration method is used in industry as a separation process in the purification and concentration of macromolecular and protein solutions. The use of ultrafiltration in the industry is quite common (such as in the dairy industry, the food industry, and the pharmaceutical industry [2]). Membranes made of organic polymers are used in ultrafilters. Polyamide, polysulfones, and cellulose are often used as polymers. Ultrafiltration systems are classified according to the membrane’s structure, morphology, and size [1]. The attention of ultrafiltration in a sample preparation method was demonstrated at the beginning of 2005. It has been used frequently in the Human Metabolome Project [14]. This project has been used in nontargeted metabolomics approaches (for targeted and especially untargeted metabolomics, where the method usually does not need to be selective to some specific metabolite) [15]. Also, some applications of UF in metabolome analysis of biological materials are summarized by Vuckovic [16].

In a general article review, it is obvious which there have been confined data over the past decade using ultrafiltration like a sample preparation method for pharmaceutical analysis. These articles focus specifically on analyzing of unbound (free) API concentrations in plasma samples. Lately, Kratzer et al. [17] published work on detecting unbound plasma concentrations of tazobactam and ceftolozane. This article analyzed with HPLC-UV using the ultrafiltration sample preparation technique. Moreover, the procedure applied in this study was similar to that applied in previous studies [18].

There is no DS analysis from plasma by ultrafiltration method in the literature. However, various pharmaceuticals such as carbamazepine, diclofenac, naproxen are retained by nanofiltration membranes in wastewater treatment plants [19].

In pharmaceutical analyses, HPLC is a widely used chromatographic separation technique [20–27]. However, the most critical step in the pharmaceutical analysis is the purification of the sample to prevent interference in the analysis. Researchers are still developing automated and easy-to-follow procedures to save time and chemicals used. Most researchers prefer common sample preparation procedures such as precipitation of proteins via organic solvents, liquid-liquid, and solid-phase extraction. At the same time, the ultrafiltration technique offers researchers a one-step and easily applicable sample preparation technique from biological materials [28].

Ultrafiltration-based sample preparation for the determination of DS in human plasma samples before HPLC-UV analysis was successfully applied in the present study. Since the ultrafiltration method acts as a sieve that filters the proteins, it is an effective method that can be used to measure the free concentration of APIs in plasma samples [17, 29]. The drug molecules bound to albumin in the blood remain unfiltered, and the unbound drug molecules that transfer to the membrane can be analyzed. Commercial and selectively existing sample preparation procedures such as QUECHERS, solid-phase extraction, and liquid-phase extraction have dominated the pharmaceutical analysis through technological improvements and utilized sample preparation techniques for the last twenty years [30, 31]. There have been limited reports to analyze the total concentration of APIs through ultrafiltration-based sample preparation [32].

Pharmacokinetics is a subbranch of pharmacology that examines the processes of absorption, distribution, transformation, and disposal of drugs into the body by establishing mathematical models. In pharmacokinetic studies, bio-pharmaceutical analysis is critical in examining the behavior of APIs in biological systems. Therefore, researchers aim to analyze the concentration of drugs in various conditions at different time rates. Protein binding can affect drug activity in one of two ways; the first changes the effective concentration of the drug, the latter changes the rate of elimination of the drug. Drugs are mainly bound to plasma proteins such as albumin, alpha-1-acid glycoprotein, lipoproteins [33]. Drugs bind to plasma proteins depending on their specific drug’s affinity. DS is a member of NSAIDs, and it binds to plasma proteins (mainlyalbumin) at a rate of 99.7%, as mentioned above [12]. An alternative and more straightforward approach, protein precipitation, is used to release protein-bound drugs. Methanol denatures proteins by acting on the bonds that hold parts of the protein folding. Various separation techniques of unbound drug fractions are known, such as ultrafiltration, ultracentrifugation, and equilibrium dialysis. Since the primary shortcoming of ultrafiltration is the analysis of free drug concentration on direct filtration of plasma samples, we used a protein precipitation agent, methanol, before the filtration procedure. In general, protein precipitation is required to release drugs fully, but shows that filtration is critical to minimize matrix effects. Using 1 : 1 (v/v) human plasma-methanol allowed us to reach acceptable recovery values for DS in our experimental conditions. Thus, it might be discussed if the researchers could employ ultrafiltration, a nonselective sample preparation technique, in pharmacokinetic studies. Precipitation of proteins combined with ultrafiltration could allow researchers an easy to apply one-step sample preparation without any variation factor depending on the analyst’s experience. In addition, ultrafiltration-based sample preparation could allow researchers to prepare 96 samples simultaneously while using a suitable centrifuge for this purpose. Compared with solid-phase extraction, it can leave the analyst independent and save time since it does not require any buffer preparation or washing steps. In our experiments, we injected more than a hundred samples, and the column backpressure was stable during these analyzes. When we compared the chromatograms for protein-free supernatant after ultrafiltration and before ultrafiltration, we did not recognize any significant differences. However, we realized that the direct injection of supernatant without ultrafiltration caused an increased back pressure in HPLC analysis after a few dozens of injections. Our previous experiences also support this critical effect on column lifetime. On the other hand, using an individual ultrafiltration device for every single sample may cause a dramatic effect on total analysis costs. In this point, it should be mentioned that the ultrafiltration-based sample preparation process presented in this study does not need any optimization procedure and researchers should consider the HPLC separation of the API from baseline after ultrafiltrated. The developed method and the ultrafiltration sample preparation step were compared with the previous methods published on diclofenac analysis from plasma using conventional sample preparation steps. Type of extraction, sample preparation solvents used, LOD, LOQ, and recovery values are presented and compared in Table 3 [34–39].

According to Table 3, the lowest LOD value belongs to the developed method. While the LOQ value of this method is the same in only one of the compared methods, it is higher in other methods. In other words, the sensitivity of the developed method, which is one of the validation parameters, is higher. In addition, an LC-Tandem MS device was used in one of the comparison methods. Although the sensitivity of this device is generally known to be higher than HPLC, the developed method was found to be more sensitive. However, the recovery value, which is another validation parameter of the developed method, is higher than other methods (especially the solid phase extraction method).

When the developed method is compared in terms of time and cost, it may seem disadvantageous at first glance, but when it is examined in general, it is not. When the methods are compared, one ultrafilter, one solid phase extraction cartridge, and various solvents are used for each sample. The cost per sample appears to be lower in liquid-liquid extraction. However, during these analyses, the column backpressure was stable even though we ultimately injected more than a hundred samples in ultrafiltration. This means that the ultrafiltration method increases the column life and automatically reduces the cost. While the extraction steps are repeated to increase the efficiency of liquid-liquid and solid-phase extraction, there is no repetition in the ultrafiltration method.

In short, the advantage of the developed method is high recovery and sensitivity while low cost and time-consuming.

## 4. Conclusion

The sample preparation step is the essential point for pharmaceutical analysis to clean up the sample and avoid interferences’ peaks. Although ultrafiltration is not a novel sample preparation technique to overcome all the negative impacts of a complex biological matrix, it has mainly found itself a place in proteomics and metabolomics studies. The proposed methodology using ultrafiltration in this study can be an example of the feasibility of this unique technique on biopharmaceutical analysis. It can be adopted to pharmacokinetic studies requiring the analysis of lots of samples. The acceptable recovery results of DS, which is very eager to bind the proteins, can lead chromatographers to employ the workflow presented in this study on further studies. In addition, it increases the column life by reducing the counter pressure in HPLC in sample injection prepared by ultrafiltration. It will provide convenience for researchers by preventing colon changes during analysis.

## Figures and Tables

**Table 1:** Linearity of the developed method (n = 6).

Regression equation for the final concentration (4x diluted) *	y = 17.3936x + 0.3390
Standard error of intercept	0.0143
Standard error of slope	0.2027
Regression coefficient (R^2^)	0.9991
Range for the final concentration (4x diluted) (μg mL^−^^1^)	0.025–0.5
The range for the targeted concentration (μg mL^−^^1^)	0.1–2.0
Number of data points	7
LOQ for the final concentration (μg mL^−^^1^)	0.020
LOD for the final concentration (μg mL^−^^1^)	0.006

* Based on six calibration curves where y: peak area ratio of DS to IS and x: final concentration of DS as μg mL^−1^.

**Table 2:** Analysis of spiked plasma samples (n = 6).

Added DS into the blank plasma samples	0.0500 μg mL^−1^		0.1125 μg mL^−1^		0.2500 μg mL^−1^	
	*intraday*	*interday*	*intraday*	*interday*	*intraday*	*interday*
Found DS in the blank plasma samples^a^	0.0476 ± 0.0024 μg mL^−^^1^	0.0495 ± 0.0021 μg mL^−^^1^	0.1161 ± 0.0063 μg mL^−^^1^	0.1136 ± 0.0068 μg mL^−^^1^	0.2467 ± 0.0093 μg mL^−^^1^	0.2383 ± 0.0068 μg mL^−^^1^
Bias of the analysis^b^	−4.77	−0.94	3.23	0.93	−1.31	−4.70
Relative standard deviation of the results %	4.96%	4.19%	5.40%	5.99%	3.79%	2.86%
Recovery %	98.99%	95.21%	100.21%	102.48%	94.37%	99.26%

a Found: mean ± standard error (n = 6),

b Bias: [(Found-Added)/Added] × 100

**Table 3:** Comparison of DS analysis from plasma with different extraction techniques.

Analyte	Method	Extraction type	Mobile phase	LOD/LOQ (μg mL^−1^)	Recovery
DS	Developed HPLC	Ultrafiltration	Acetonitrile : phosphate buffer (pH 3.0, 10 mM) (70 : 30 v/v)	0.006/0.020	98.27%
	Reported HPLC [34]	Solid Phase Extraction	MeOH : Water (pH 3.3) (63 : 37 v/v)	0.042/1.000	89.8%
	Reported HPLC [35]	Solid Phase Extraction	Acetonitrile : phosphate buffer containing 0.1% trifluoroacetic acid (pH 7.0, 20 mM) (35 : 65 v/v)	– /0.075	93.8–101.2%
	Reported LC-Tandem MS [36]	Solid Phase Extraction	Acetonitrile : water containing 0.2% acetic acid (80 : 20 v/v)	– /0.025	79%
	Reported HPLC [37]	Liquid-Liquid Extraction	Acetonitrile : water (pH 3.3) (50 : 50 v/v)	0.015/0.025	110%
	Reported HPLC [38]	Liquid-Liquid Extraction	Acetonitrile : water (pH 4) (55 : 45 v/v)	0.01/0.02	-
	Reported HPLC [39]	Liquid-Liquid Extraction	Acetonitrile : water (pH 3.3) (50 : 50 v/v)	0.00895/0.02712	98.75–99.32%
